# Electron Detachment Dissociation for Top-Down Mass Spectrometry of Acidic Proteins

**DOI:** 10.1002/chem.201003709

**Published:** 2011-03-23

**Authors:** Barbara Ganisl, Taras Valovka, Markus Hartl, Monika Taucher, Klaus Bister, Kathrin Breuker

**Affiliations:** [a] Institute of Organic Chemistry and Center for Molecular Biosciences Innsbruck (CMBI), University of InnsbruckInnrain 52a, 6020 Innsbruck (Austria), Fax: (+43) 512-507-2892 E-mail: kathrin.breuker@uibk.ac.at; [b] Institute of Biochemistry and Center for Molecular Biosciences Innsbruck (CMBI), University of InnsbruckPeter-Mayr-Strasse 1a, 6020 Innsbruck (Austria)

**Keywords:** acidic proteins, electron detachment dissociation, FT-ICR, mass spectrometry, sequencing

## Abstract

Electron detachment dissociation (EDD) is an emerging mass spectrometry (MS) technique for the primary structure analysis of peptides, carbohydrates, and oligonucleotides. Herein, we explore the potential of EDD for sequencing of proteins of up to 147 amino acid residues by using top-down MS. Sequence coverage ranged from 72 % for Melittin, which lacks carboxylic acid functionalities, to 19 % for an acidic 147-residue protein, to 12 % for Ferredoxin, which showed unusual backbone fragmentation next to cysteine residues. A limiting factor for protein sequencing by EDD is the facile loss of small molecules from amino acid side chains, in particular CO_2_. Based on the types of fragments observed and fragmentation patterns found, we propose detailed mechanisms for protein backbone cleavage and side chain dissociation in EDD. The insights from this study should further the development of EDD for top-down MS of acidic proteins.

## Introduction

The unique radical-ion chemistry involved in electron capture dissociation (ECD)[1] produces fragment ions from protein backbone cleavage that provide detailed sequence information in top-down mass spectrometry (MS) experiments,[2] including the identification and localization of post-translational modifications (PTMs).[2b, 3] ECD requires precursor ions carrying multiple positive charges, typically [*M*+*n* H]^*n*+^ ions formed by electrospray ionization (ESI).[4] Acidic proteins, however, are more readily ionized under ESI operated in negative ion mode, which generally produces [*M*−*n* H]^*n*−^ ions.[5] Electron detachment dissociation (EDD),[6] introduced by Zubarev and co-workers, also involves radical ion chemistry and can be applied to [*M*−*n* H]^*n*−^ ions. It has been shown that EDD provides information on the primary structure of peptides,[6b, 7] carbohydrates,[8] and oligonucleotides.[9] In EDD, irradiation of multiply deprotonated ions with >10 eV electrons results in electron detachment, which produces radical [*M*−*n* H]^(*n*−1)−•^ ions that can undergo backbone dissociation.[9e,f] EDD is carried out in ion-trapping instruments operated under high vacuum conditions (≈10^−10^ mbar) in which electrons have sufficiently long mean free paths, that is, Fourier transform ion cyclotron resonance (FT-ICR)[10] mass spectrometers. Recently, negative electron transfer dissociation (NETD)[11] was introduced as an alternative to EDD that can be implemented in Paul-type ion-trap instruments operated at higher pressures (≈10^−3^ mbar). In NETD, the [*M*−*n* H]^*n*−^ ions from ESI collide with and transfer an electron to radical cations, such as Xe^+•^ or fluoranthene cations (C_16_H_10_^+•^). A related method is electron-photodetachment dissociation (EPD),[12] in which radical [*M*−*n* H]^(*n*−1)−•^ ions are formed by electron detachment from [*M*−*n* H]^*n*−^ ions upon laser irradiation with UV photons. The [*M*−*n* H]^(*n*−1)−•^ ions from EPD were found to be rather stable, showing no or little fragmentation on the timescale of the experiment, but extensive dissociation into products similar to those from EDD was observed if the radical [*M*−*n* H]^(*n*−1)−•^ ions were subjected to low-energy collisional activation.[12a,b]

Yet another MS variant of unimolecular dissociation based on electron–ion interactions is electron-induced dissociation (EID),[13] which is derived from electron impact excitation of ions from organics.[14] In EID, the impacting electrons with energies ranging from ≈5 to 70 eV[13, 14b] excite the ions under study to a degree sufficient for unimolecular dissociation, but electrons are neither captured by nor detached from the even-electron [*M*+*n* H]^*n*+^ or [*M*−*n* H]^*n*−^ precursor ions. Based on the observation that EID of sulfated glycosaminoglycan [*M*−H]^−^ ions produced singly charged odd-electron fragment ions, which obviously cannot arise from electron detachment, and that vibrational excitation of the [*M*−H]^−^ ions by infrared multiphoton dissociation (IRMPD) did not produce odd-electron fragment ions, Amster and co-workers have suggested that EID involves ion activation by electronic excitation.[13a] Because electron energies for EDD are generally higher than 10 eV, some of the products in EDD spectra may actually result from EID processes.

Our motivation for this study was to explore the potential of EDD for top-down mass spectrometry of acidic proteins, utilizing the high mass resolving power and accuracy of FT-ICR instruments. We have studied here the unimolecular dissociation by EDD of highly charged [*M*−*n* H]^*n*−^ ions of Melittin, Ubiquitin, Ferredoxin, and an acidic 147 amino acid residue construct (BASP1(Δ121–216)) derived from BASP1 (brain acid soluble protein), with calculated pI values[15] of 12.02, 6.56, 3.88, and 4.61, respectively. Surprisingly, the highest yield of sequence-informative fragment ions from protein backbone cleavage was obtained for the most basic protein, Melittin, whereas sequence coverage was smallest for the acidic proteins, Ferredoxin and BASP1(Δ121–216). We rationalize our results on the basis of thermochemical considerations and radical-ion chemistry.

## Results and Discussion

**Figure 1:**
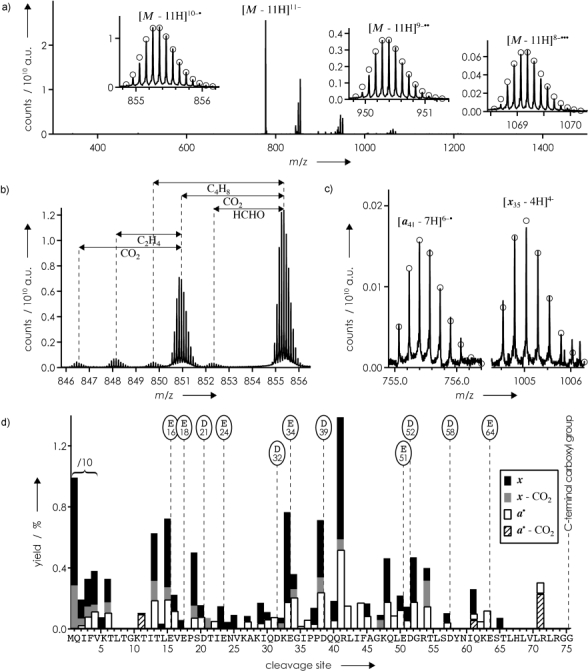
a) Mass spectrum of products from EDD of [*M*−11 H]^11−^ ions of Ubiquitin electrosprayed from a 1 μm solution (1:1 H_2_O/CH_3_OH, 0.1 % v/v DBU, pH 10.5); insets: highlighted peaks of oxidized molecular ions. b) The *m*/*z* region illustrating small neutral losses from [*M*−11 H]^10−•^ ions. c) The *m*/*z* regions showing peaks of complementary [***a***_41_−7 H]^6−•^ and [***x***_35_−4 H]^4−^ ions; calculated isotopic profiles (see Table 1) are displayed as ○. d) The site-specific yield of products from backbone cleavage vs. backbone cleavage site (ion yields for cleavage sites 1–4 are shown reduced by a factor of 10 for scaling reasons); acidic residue locations are indicated as dashed lines.

**Figure 2:**
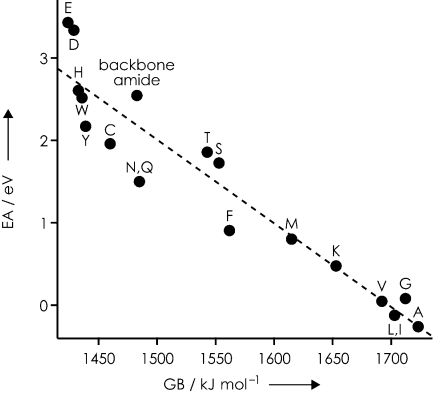
Electron affinity of X^•^ vs. gas-phase basicity of X^−^ for compounds XH as models for amino acid side chains and backbone amide, as indicated.

Figure 1a shows a mass spectrum of products from electron detachment dissociation of [*M*−11 H]^11−^ ions of Ubiquitin. Three different classes of EDD products were observed: 1) oxidized molecular ions, that is, [*M*−11 H]^10−•^, [*M*−11 H]^9−••^, [*M*−11 H]^8−•••^, and [*M*−11 H]^7−••••^ (Figure 1a); 2) products from small neutral losses from oxidized molecular ions (Figure 1b); and 3) ***a***^•^ and ***x*** fragment ions from protein backbone cleavage (Figure 1c). The radical [*M*−*n* H]^(*n*−*m*)−*m*•^ ions formed by detachment of up to three electrons from molecular ions constituted 34 % of all EDD products. Mass values for small-molecule losses (Figure 1b) from oxidized molecular ions can be assigned accurately by taking advantage of the high mass resolving power and accuracy of the FT-ICR mass spectrometer. For this purpose, the most abundant isotopes of the [*M*−11 H]^11−^, [*M*−11 H]^10−•^, [*M*−11 H]^9−••^, and [*M*−11 H]^8−•••^ ions of Ubiquitin were used for internal calibration of the EDD spectrum (Table 1).

**Table 1:** Analysis of the mass spectrum in Figure 1

exptl *m*/*z*[a]	assignment	calcd *m*/*z*[a]	error [ppm]
777.59561[b]	[*M*−11 H]^11−^	777.59549	−0.15
855.35502[b]	[*M*−11 H]^10−•^	855.35498	−0.04
950.39460[b]	[*M*−11 H]^9−••^	950.39437	−0.25
1069.19341[b]	[*M*−11 H]^8−•••^	1069.19359	0.17
852.35401	[*M*−11 H−HCHO]^10−•^	852.35393	−0.10
850.95601	[*M*−11 H−CO_2_]^10−•^	850.95600	−0.01
849.74803	[*M*−11 H−C_4_H_8_]^10−•^	849.74872	0.82
848.15275	[*M*−11 H−CH_2_CHCOOH]^10−•^	848.15287	0.14
846.55712	[*M*−11 H−2CO_2_]^10−•^	846.55702	−0.12
755.39835	[***a***_41_−7 H]^6−•^	755.39824	−0.14
1005.03984	[***x***_35_−4 H]^4−^	1005.03968	−0.16

[a] *m/z* values of the most abundant isotopic peak.

[b] Used for internal calibration.

The predominant small-molecule loss from oxidized molecular ions of Ubiquitin (Figure 1b) corresponds to a Δ*m* value of 43.990 Da, which can be assigned to loss of CO_2_, presumably from aspartic and glutamic acid side chains and the C terminus. Collisionally activated dissociation (CAD) of [*M*−11 H]^11−^ ions of Ubiquitin did not produce [*M*−11 H−CO_2_]^11−^ (see Figure S1 in the Supporting Information), and only 0.7 % of the [*M*−11 H]^11−^ precursor ions showed loss of CO_2_ in EDD. Therefore, the majority (>99 %) of [*M*−11 H−CO_2_]^10−•^ ions in EDD must have resulted from electron detachment, rather than being formed by EID. Other small neutral losses, although of considerably smaller abundance, correspond to Δ*m* values of 30.010, 56.070, and 72.023 Da and elemental compositions of C_1_H_2_O_1_, C_4_H_8_, and C_3_H_4_O_2_, respectively. Again, these compositions are consistent with losses from side chains, that is, HCHO from serine, C_4_H_8_ from leucine or isoleucine, and CH_2_CHCOOH from glutamic acid. Importantly, CO_2_, HCHO, C_4_H_8_, and CH_2_CHCOOH are even-electron species; as a consequence, the radical site must be located on the large complementary protein fragment after side chain dissociation (see Scheme S1 in the Supporting Information). The observation of [*M*−11 H−2 CO_2_]^10−•^ and [*M*−11 H−CO_2_−C_4_H_8_]^10−•^ ions (Figure 1b) is consistent with detachment of two electrons followed by electron capture. Alternatively, radical migration within the [*M*−11 H−CO_2_]^10−•^ ions could result in secondary loss of CO_2_ or C_4_H_8_. As shown below, deprotonation of and electron detachment from leucine or isoleucine side chains is energetically unfavorable, so that radical migration appears the more probable pathway for loss of C_4_H_8_.

The yield of products from small (<100 Da) neutral losses from oxidized molecular ions was 43 %, thus a total of 77 % of all EDD products were formed by processes not involving protein backbone cleavage. To put this another way, only 23 % of the products in EDD of [*M*−11 H]^11−^ ions of Ubiquitin were sequence-informative fragment ions. This value is rather small compared to the approximately 60 % yield of backbone fragments in ECD of multiply protonated [*M*+11 H]^11+^ ions of Ubiquitin.[1e] It has been shown that noncovalent bonding in gaseous [*M*+*n* H]^*n*+^ Ubiquitin ions (*n*=5–8) can prevent fragment-ion separation in ECD and, therefore, can significantly reduce fragment ion yields; ECD of the [*M*+5 H]^5+^ ions gave no separated backbone fragments at all.[1e] However, collision cross sections[16] and ECD data[1e] indicate that the more highly charged [*M*+*n* H]^*n*+^ ions of Ubiquitin (*n*>10) have extended structures. Collision cross sections for [*M*−*n* H]^*n*−^ ions of Ubiquitin are not available, but those of Cytochrome c suggest that for highly charged protein ions (*n*>10 for Ubiquitin), the collision cross sections do not depend on ion polarity.[17] Moreover, to minimize possible intramolecular noncovalent interactions, the [*M*−11 H]^11−^ Ubiquitin ions were activated by energetic collisions (27.5 eV laboratory frame energy) with Ar gas prior to EDD. From the above considerations, it appears that the small (23 %) fragment-ion yield in EDD of [*M*−11 H]^11−^ ions of Ubiquitin is limited by factors other than noncovalent bonding. Similarly small fragment-ion yields of 4, 19, 20, and 22 % were observed in NETD of [*M*−9 H]^9−^, [*M*−10 H]^10−^, [*M*−11 H]^11−^, and [*M*−12 H]^12−^ ions of Ubiquitin, respectively.[18]

Unimolecular backbone dissociation in EDD of [*M*−11 H]^11−^ ions of Ubiquitin gave pairs of complementary ***a***^•^ and ***x*** fragment ions, illustrated in Figure 1c for [***a***_41_−7 H]^6−•^ and [***x***_35_−4 H]^4−^. Note that the charges of [***a***_41_−7 H]^6−•^ and [***x***_35_−4 H]^4−^ add up to the charge of [*M*−11 H]^10−•^, and, within experimental error (Δ*m*=0.18 ppm), the sum of the measured monoisotopic mass values of [***a***_41_−7 H]^6−•^ and [***x***_35_−4 H]^4−^ ((755.06412×6) Da+(1004.53851×4) Da=8548.5388 Da) give the mass value of [*M*−11 H]^10−•^ ((854.85372×10) Da=8548.5372 Da). This data is in agreement with the proposed mechanism for ***a***^•^ and ***x*** ion formation in EDD.[6b, 11c, 19] For all proteins studied here, the even-electron fragment ions were more abundant than their radical complements, with [***x***]/[***a***^•^]≈4:1 in EDD of [*M*−11 H]^11−^ ions of Ubiquitin. For EDD of doubly deprotonated peptide ions, Zubarev and co-workers have reported that different peptides give different [***x***]/[***a***^•^] ratios.[6] However, because one of the charges is neutralized in EDD of [*M*−2 H]^2−^ ions, only one of the two resulting complementary fragments (either ***a***^•^ or ***x***) carries charge and can be detected in the mass spectrometer, so ***a***^•^ and ***x*** ion signals in the EDD spectrum of [*M*−2 H]^2−^ ions do not necessarily reflect relative ***a***^•^ and ***x*** product abundances. In contrast, the majority of ***a***^•^ and ***x*** fragment ions from EDD of the highly charged [*M*−11 H]^11−^ ions of Ubiquitin carry charge and can thus be detected. The observed [***x***]/[***a***^•^] ratio of about 4:1 is then consistent with a significantly lower stability of the radical ***a***^•^ ions when compared to that of the even-electron ***x*** ions. Loss of CO_2_ from ***a***^•^ and ***x*** ions was 6 and 19 % relative to all ***a***^•^ and ***x*** ions, respectively. However, other products from secondary fragmentation of ***a***^•^ ions were not observed, probably because they dissociate via multiple channels; the resulting secondary fragments would then give rise to signals with too small signal-to-noise ratio for detection.

The extensive CO_2_ loss discussed above suggests that electron detachment from negatively charged sites is a major process in EDD of [*M*−*n* H]^*n*−^ protein ions. However, can we exclude electron detachment from neutral sites? Electron energies used for EDD in this study (24–28 eV) are far higher than experimentally determined ionization energies (IE) of uncharged amino acids that lie between 8 and 10 eV,[20] as well as calculated vertical IEs ranging from 7.1 eV for tryptophan to 9.9 eV for serine.[21] Ionization energies of uncharged amino acid residues are unknown, but data for glycine (adiabatic IE=8.9 eV, vertical IE=10.0 eV), *N*-acetylglycine (adiabatic IE=9.4 eV, vertical IE=9.8 eV), and glycine methyl ester (adiabatic IE=9.1 eV, vertical IE=9.8 eV)[20a] suggest that amino acid residue IEs differ from IEs of amino acids by less than 1 eV. Zubarev and co-workers have used experimental data from electron ionization of multiply protonated peptides for extrapolation of vertical ionization energies for five different neutral peptides to (9.8±0.3) eV.[22] The above data provide a conservative estimate of 7 to 10 eV for ionization energies of uncharged amino acid residues. Although the impacting electrons will lose some of their kinetic energy of 24 to 28 eV on approaching the multiply charged protein anions as a result of Coulombic repulsion, the remaining electron kinetic energy should be sufficient to effect ionization at neutral sites.

Ionization energies of deprotonated amino acid residues are generally not known. However, electron affinities (EA) of some organic radicals, X^•^, have been evaluated and compiled in the NIST Standard Reference Database.[23] Because the electron attachment reaction, X^•^+e^−^→X^−^, can be rearranged into the “ionization reaction” of the organic anion, X^−^−e^−^→X^•^, it follows that EA(X^•^)=IE(X^−^). Note that the latter reaction is in fact a charge neutralization reaction, but the term “ionization energy” is used herein to indicate the energy required to remove an electron from X^−^, in analogy to ionization of neutrals, X−e^−^→X^+•^. For example, CH_3_CH_2_O^•^ has an electron affinity of 1.73 eV, which at the same time is the IE of the corresponding anion, CH_3_CH_2_O^−^. We have searched the NIST Standard Reference Database for compounds XH that best resemble the common amino acid side chains and the backbone amide, and for which the relevant energetic and thermochemical data are available. The electron affinities of the corresponding radicals, EA(X^•^), and the gas-phase basicities of the corresponding anions, GB(X^−^), retrieved from this search are listed in Table 2 and shown in Figure 2. The EA(X^•^) values show a near-linear negative correlation with GB(X^−^) because either the radical X^•^ or the corresponding anion X^−^ can be stabilized, but not both. From the data in Table 1, the ionization energies of deprotonated amino acid side chains and backbone amides can be estimated to generally not exceed 3.5 eV, which is substantially smaller than the estimated IE values for neutral amino acid residues of 7 to 10 eV. For energetic reasons, it is reasonable to assume that electrons are primarily detached from negatively charged sites in EDD of proteins.

**Table 2:** GB(X^−^) and EA(X^•^) of compounds XH as models for functional groups in proteins as indicated[a]

GB(X^−^) [kJ mol^−1^]	X^•^	EA(X^•^) [eV]	model
1424	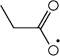	3.43	Glu
1429	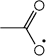	3.34	Asp
1433^[b]^	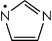	2.61^[b]^	His
1436	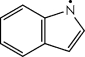	2.52	Trp
1439	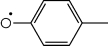	2.17	Tyr
1460	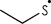	1.96	Cys
1483	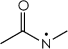	2.55	backbone amide
1485	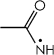	1.50	Asn, Gln
1543	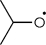	1.86	Thr
1553	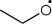	1.73	Ser
1562	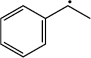	0.91	Phe
1615	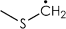	0.80	Met
1653	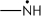	0.48	Lys
1692	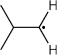	0.05	Val
1703	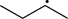	−0.12	Leu, Ile
1712	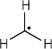	0.08	Gly
1723	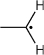	−0.26	Ala

[a] All data is from reference [23], except where indicated.

[b] Data from reference [24].

Which sites in [*M*−*n* H]^*n*−^ anions from ESI are deprotonated? The gas-phase basicity values in Table 2 indicate that negative-charge locations in gaseous, multiply deprotonated peptides and proteins are by preference at acidic side chains, that is, glutamic and aspartic acid residues and the C terminus. Accordingly, the number of carboxylic acid groups in Ubiquitin of 12 (6 E, 5 D, C terminus) is close to the maximum number of charges (13) of [*M*−*n* H]^*n*−^ ions from ESI in negative-ion mode (see Figure S2 in the Supporting Information).

It has been suggested that EDD backbone cleavage should occur near acidic residues and be disfavored near hydrophobic residues.[6b] However, the site-specific yield of fragment ions from backbone cleavage in EDD of [*M*−11 H]^11−^ ions of Ubiquitin shows no clear correlation with acidic residue location within the sequence (Figure 1d). The highest yield of fragment ions was obtained from cleavage near the N terminus (cleavage sites 1–4) and cleavage site 41, but the 1–15 and 39–49 segments completely lack acidic residues. Moreover, fragment ions from backbone cleavage next to residues E24, D58, E64, and the C terminus are rather low in abundance, or even absent. Also in contrast to the above suggestions,[6b] backbone fragmentation was observed in the 43–47 segment that consists only of hydrophobic residues. Our EDD data for Ubiquitin suggest that carboxylic acid groups (D, E, C terminus) may not necessarily be required for EDD backbone cleavage in proteins and that backbone cleavage is not always disfavored near hydrophobic residues.

To further test our hypothesis, we studied EDD of the small protein Melittin, which completely lacks carboxylic acid functionalities. According to the gas-phase basicity values in Table 2, deprotonation of Melittin, which has no D, E, H, Y, or C residues, should be favored at W, N, Q side chains and backbone amides. The yield of oxidized molecular ions, products from small neutral losses from oxidized molecular ions, and fragment ions from backbone cleavage in EDD of [*M*−4 H]^4−^ ions of Melittin was 51, 6, and 43 %, respectively. Small-molecule losses from oxidized molecular ions in EDD of Mellitin [*M*−4 H]^4−^ ions correspond to Δ*m* values of 44.023 and 129.054 Da, which is consistent with loss of CH_3_CHO from threonine and C_9_H_7_N from tryptophan (see Scheme S1 in the Supporting Information). Importantly, the loss of small molecules from [*M*−*n* H]^(*n*−*m*)−*m*•^ ions is reduced from 43 % in EDD of Ubiquitin to 6 % in EDD of Melittin, which substantiates our above hypothesis and suggests that loss of CO_2_ is an independent fragmentation channel in EDD of peptides and proteins.

**Figure 3:**
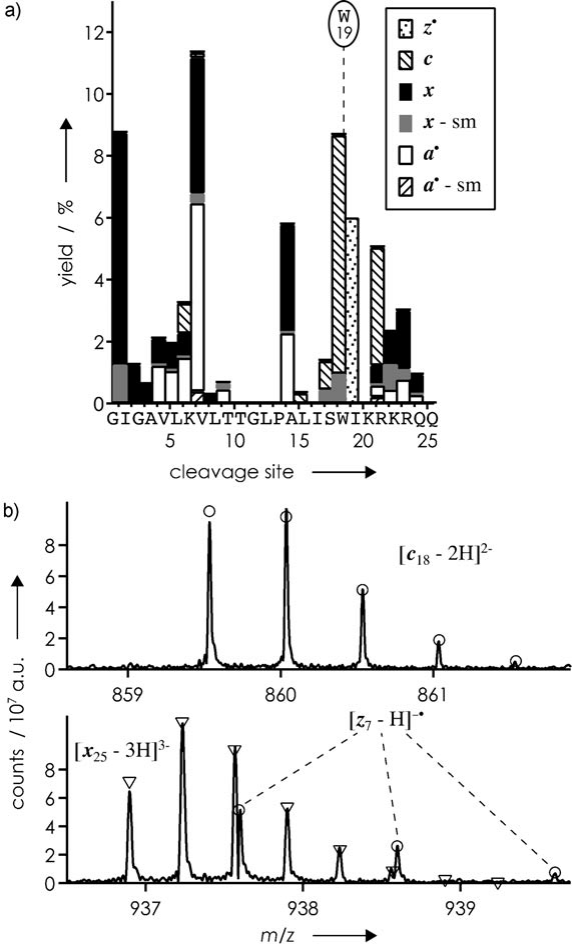
a) The site-specific yield of products from backbone cleavage in EDD of [*M*−4 H]^4−^ ions of Melittin electrosprayed from a 1 μm solution (1:1 H_2_O/CH_3_OH, 0.5 % v/v DBU, pH 12.5); sm=small (<100 Da) molecule. b) The *m*/*z* regions showing peaks of [***c***_18_−2 H]^2−^ and [***z***_7_−H]^−•^ ions; calculated isotopic profiles are displayed as ○. The calculated isotopic distribution for [***x***_25_−3 H]^3−^ ions is shown as ▿.

The highest yield of ***a***^•^ and ***x*** fragments from backbone cleavage was found next to K7, which is framed by three and two hydrophobic amino acid residues on its C- and N-terminal sides, respectively (Figure 3a). Other abundant ***a***^•^ and ***x*** ions are from backbone cleavage next to the N terminus, P14, and the basic region near the C terminus (residues 21–24). These findings for EDD of Melittin generally agree with those for EDD of Ubiquitin, which gave the highest yield of fragment ions from backbone cleavage near the N terminus (cleavage sites 1–4) and next to R41.The EDD data for Ubiquitin and Melittin suggest that basic residues can actually facilitate ***a***^•^ and ***x*** ion formation in EDD.

**Scheme 1:**
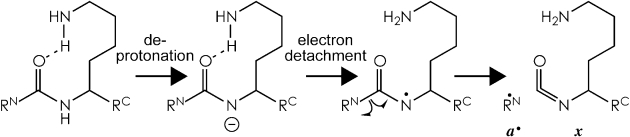
Proposed structure of basic residues, shown here for lysine, forming a hydrogen bond to an adjacent backbone oxygen, which facilitates amide deprotonation. Electron detachment from the negatively charged site results in ***a***^•^ and ***x*** ion formation by protein backbone cleavage; R^N^ and R^C^ stand for N-terminal and C-terminal residues, respectively.

Scheme 1 provides a possible rationale for the preferred fragmentation into ***a***^•^ and ***x*** fragment ions next to basic residues. It implies the formation of a hydrogen bond between a basic residue and an adjacent backbone amide oxygen during the ESI process. This structural arrangement would facilitate amide deprotonation by acidifying the amide hydrogen. Electron detachment from the negatively charged site would then result in formation of ***a***^•^ and ***x*** fragment ions, as shown in Scheme 1. Note that direct proton transfer from the backbone amide (estimated GB of corresponding anion=1483 kJ mol^−1^, see Table 2) to an uncharged basic side chain is energetically unfavorable by at least 468 kJ mol^−1^ because the gas-phase basicities of K, H, and R are 928, 936, and 992 kJ mol^−1^, respectively, and the gas-phase basicities of di-, tri-, and pentapeptides containing K, H, and R do not exceed 1015 kJ mol^−1^.[25]

**Scheme 2:**
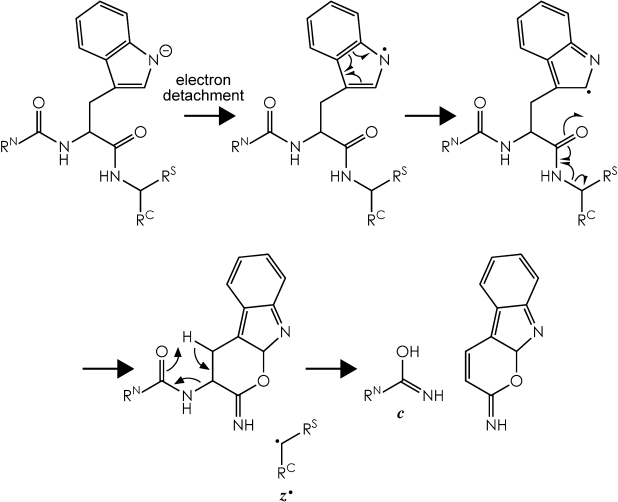
Proposed mechanism for ***c*** and ***z***^•^ ion formation by protein backbone cleavage next to tryptophan; R^N^, R^C^, and R^S^ stand for N-terminal, C-terminal, and side chain residues, respectively. The tricyclic neutral species formed in the last reaction step has a calculated mass of 184.064 Da.

In addition to the ***a***^•^ and ***x*** fragment ions characteristic for EDD of multiply deprotonated peptides, products from EDD of Melittin also include ***c***- and ***z***^•^-type fragment ions (Figure 3b). The latter species are typical ECD products of [*M*+*n* H]^*n*+^ protein ions,[1a, 26] so it is surprising to find them in EDD spectra of protein anions. However, ***c*** ion formation in unimolecular dissociation of radical peptide anions[6a, 12c,d] and cations[27] has been reported. The most abundant ***c*** and ***z***^•^ fragments in EDD of Melittin [*M*−4 H]^4−^ ions are [***c***_18_−2 H]^2−^ and [***z***_7_−H]^−•^ (Figure 3b); the sum of the monoisotopic mass values (1719.0690+937.5981=2656.667 Da) of these fragments differ from that of [*M*−4 H]^3−•^ (2840.725 Da) by 184.058 Da. Judging from the data in Table 2, the tryptophan residue at position 19 actually has the highest probability for deprotonation in Mellitin [*M*−*n* H]^*n*−^ ions. A possible mechanism that accounts for the observed formation of non-complementary ***c*** and ***z***^•^ ions by way of backbone cleavage next to tryptophan residues is shown in Scheme 2. The proposed mechanism is similar to the widely accepted mechanism for ***c*** and ***z***^•^ ion formation in ECD[1a, d, 28] in that unimolecular radical-ion chemistry initiates the formation of a new bond to an amide oxygen and causes cleavage of the associated N–Cα bond.

It has been reported that EPD of Melittin [*M*−2 H]^2−^ ions also gives abundant fragment ions from N–Cα bond cleavage next to W19, although a comparison of spectral data with calculated isotopic profiles indicated that these were odd-electron ***c***_18_^−•^ and even-electron ***z***_8_^−^ ions.[12c] Radical cation formation by photodetachment of an electron from uncharged tryptophan was suggested as the first step in a possible mechanism for ***c***^•^ and ***z*** ion formation.[12c] However, considering the high probability for deprotonation of W19 in Melittin [*M*−*n* H]^*n*−^ ions, and the ionization energies of deprotonated indole (2.52 eV, see Table 2) and neutral indole (7.76 eV)[23] as model systems for deprotonated and neutral tryptophan, respectively, it is more likely that EPD at 260 nm[12c] (corresponding to 4.77 eV photon energy) involves electron detachment from deprotonated W19, just like in EDD of Melittin.

**Figure 4:**
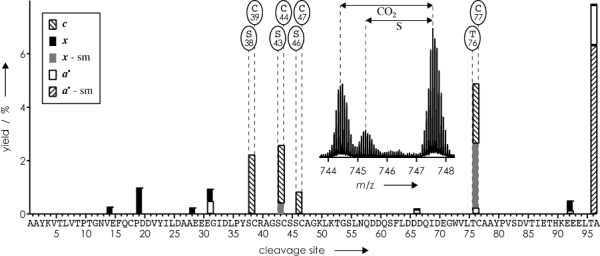
a) Site-specific yield of products from backbone cleavage vs. backbone cleavage site in EDD of [*M*−15 H]^15−^ ions of Ferredoxin electrosprayed from a 1 μm solution (1:1 H_2_O/CH_3_OH, 0.1 % v/v DBU, pH 10.5); sm=small (<100 Da) molecule. Inset: The loss of S (Δ*m*_exptl_=31.982; Δ*m*_calcd_=31.972) and CO_2_ (Δ*m*_exptl_=44.004; Δ*m*_calcd_=43.990) from [*M*−15 H]^14−•^ ions.

We also identified another acidic protein that produces ***c*** ions in EDD, that is, the cysteine-rich (C18, C39, C44, C47, and C77) protein Ferredoxin. Abundant even-electron ***c*** ions from cleavage on the N-terminal side of C39, C44, C47, and C77 were observed, whereas no products were found from backbone cleavage next to C18 (Figure 4). A common feature of residues C39, C44, C47, and C77 is that the adjacent residue on their N-terminal side carries a hydroxyl group at the Cβ carbon, whereas the residue on the N-terminal side of C18 (Q17) does not. Apparently, a hydroxyl group at the Cβ carbon on the N-terminal side of cysteine residues facilitates ***c*** ion formation. Moreover, from the data in Table 2, the side chain of cysteine has a higher probability for deprotonation than those of Q, T, or S. Electron detachment from a deprotonated cysteine side chain could then produce a cysteinyl radical. Distonic radical cations of cysteine, H_3_N^+^–CH(CH_2_S^•^)COOH, were actually found to be long-lived in the gas phase, and fragmentation by loss of ^•^COOH, CH_2_S, ^•^CH_2_SH, and H_2_S was observed only after collisional activation.[29] From this we infer that cysteinyl radical sites, formed by electron detachment from deprotonated cysteine residues, are sufficiently long-lived to allow them to adopt specific structures from which more structurally demanding fragmentation can occur. This could reduce fragmentation of cysteinyl radical side chains by loss of S (Figure 4) and favor backbone fragmentation.

**Scheme 3:**
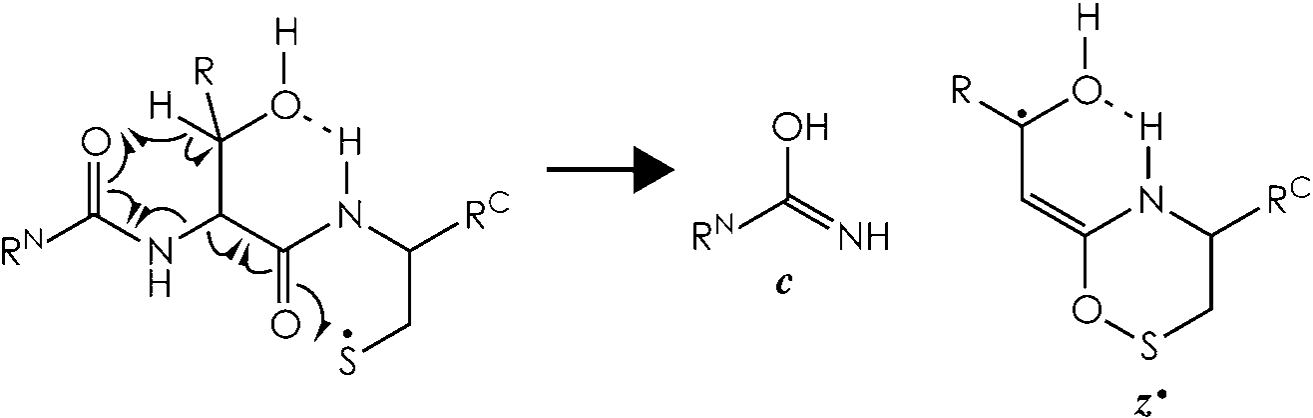
Proposed mechanism for ***c*** ion formation by protein backbone cleavage between serine or threonine and cysteine; R^N^ and R^C^ stand for N-terminal and C-terminal residues, respectively. R=H for serine and CH_3_ for threonine.

A possible mechanism for ***c*** ion formation that accounts for the above considerations is shown in Scheme 3. Here the hydroxyl group of S or T forms a hydrogen bond with the backbone NH of cysteine, thereby appropriately orienting the S or T side chain for hydrogen abstraction from the Cβ carbon by the adjacent amide oxygen. Again, the proposed mechanism is similar to the mechanism for ***c*** and ***z***^•^ ion formation in ECD;[1a, d, 28] here unimolecular radical-ion chemistry initiates the formation of two new bonds to adjacent amide oxygens. The complementary radical ***z***^•^ ions were, however, not observed, probably because these are unstable and undergo secondary dissociation. Interestingly, radical formation at cysteine and tryptophan residues by photooxidation has also been reported in solution phase experiments.[30]

**Figure 5:**
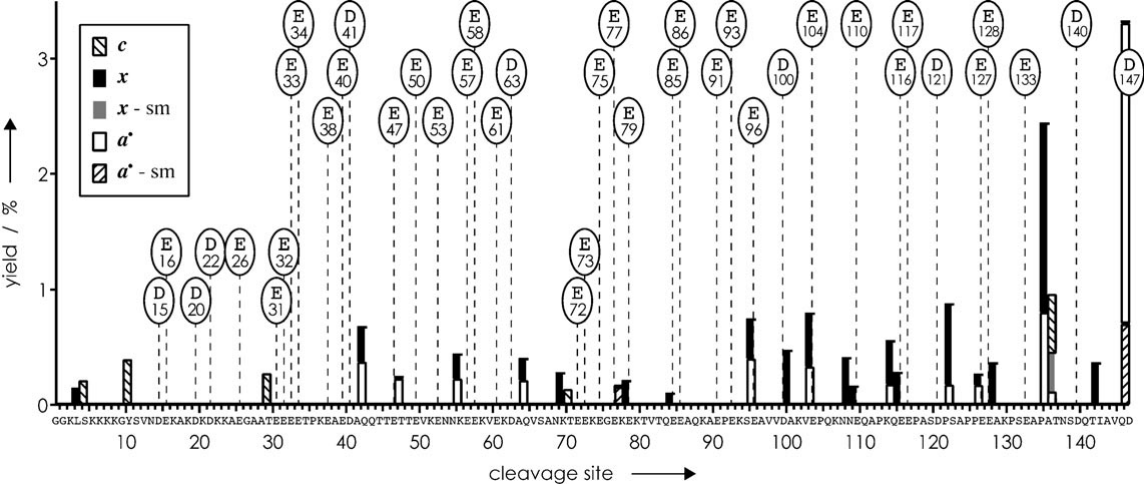
a) Site-specific yield of products from backbone cleavage vs. backbone cleavage site in EDD of [*M*−23 H]^23−^ ions of BASP1(Δ121–216) electrosprayed from a 1 μm solution (1:1 H_2_O/CH_3_OH, 0.1 % v/v DBU, pH 10.5); sm=small (<100 Da) molecule.

To extend our study to even larger proteins with an even higher relative frequency of carboxylates, we expressed and purified a recombinant protein construct derived from BASP1,[31] termed BASP1(Δ121–216) (see the Supporting Information). The yield of oxidized molecular ions, products from small neutral losses from oxidized molecular ions, and fragment ions from backbone cleavage in EDD of [*M*−23 H]^23−^ ions of BASP1(Δ121–216) was 18, 62, and 9 %, respectively; 11 % of the ions could not be assigned. The yield of fragment ions from backbone cleavage (9 %) was even smaller than that for Ferredoxin (13 %). Similar to the EDD data for Ubiquitin (Figure 1), the site-specific yield of fragment ions from EDD of [*M*−23 H]^23−^ ions of BASP1(Δ121–216) did not show a clear correlation with the location of acidic residues within the sequence (Figure 5). The highest yield of ***a***^•^ and ***x*** fragment ions was obtained from cleavage between P135 and A136, with the 134–139 region completely lacking D and E residues. On the other hand, regions with high relative frequency of D and E residues, for example, the 15–26 or 31–41 segments, did not give any products from backbone cleavage.

**Figure 6:**
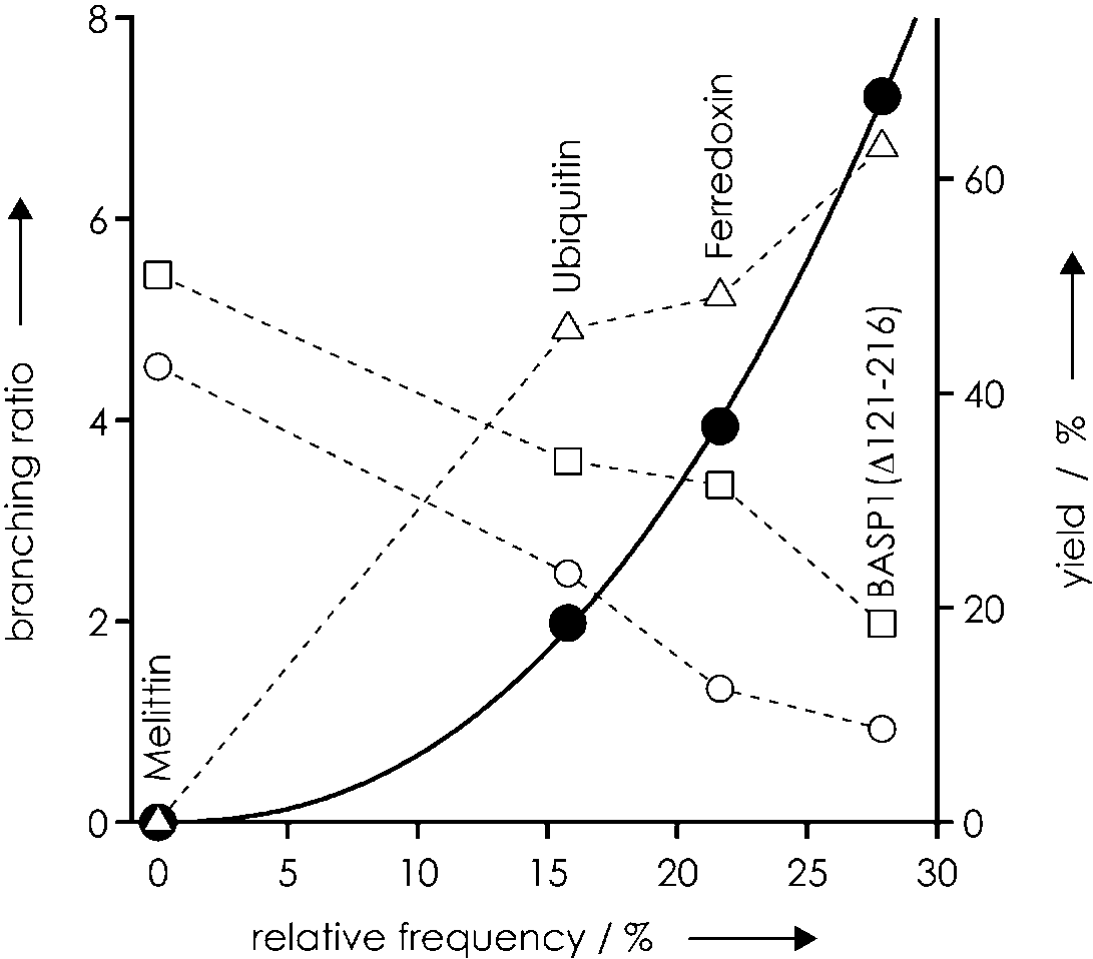
Branching ratio (•, left axis) of products from CO_2_ loss and fragments from protein backbone cleavage (***c*** and ***x*** ions) vs. relative frequency of carboxylates for each protein studied. ○, □, and ▵ represent yields (right axis) of backbone fragments (***c***+***x*** ions), oxidized molecular ions ([*M*−*n* H]^(*n*−*m*)−*m*•^), and products showing CO_2_ loss, respectively.

The idea that EDD backbone cleavage should occur near acidic residues is based on theoretical evidence, which indicates that carboxylates can form ionic hydrogen bonds with backbone amides in the gas phase.[6b, 32] Ab initio calculations suggest that electron detachment from a carboxylate that forms an ionic hydrogen bond with a backbone amide results in either loss of CO_2_, or backbone cleavage into ***a***^•^ and ***x*** fragments, with the CO_2_ loss channel being more favorable by 54 kJ mol^−1^.[6b] If these channels are competitive, as suggested by the calculations,[6b] the branching ratio of products from CO_2_ loss and fragment ions from protein backbone cleavage into ***a***^•^ and ***x*** fragments, [CO_2_ loss]/[***x***], should not depend on the number of carboxylates in the protein. However, this branching ratio is 1.98 for Ubiquitin and 8.79 for BASP1(Δ121–216). For Melittin, which does not carry any carboxylic acid functions, this branching ratio is necessarily zero. For Ferredoxin, which produced significant yields of ***c*** ions ([***c***]/[***x***]=1.53), the [CO_2_ loss]/[***x***] branching ratio is 9.96. Taking into account both ***x*** and ***c*** ions, the branching ratio [CO_2_ loss]/([***c***]+[***x***]) increases nonlinearly with the number of carboxylates normalized to the total number of amino acid residues (Figure 6, left axis). This is largely a result of increasing CO_2_ loss with increasing relative frequency of carboxylates because the yield of both backbone fragments and oxidized molecular ions decreases nearly linearly with increasing relative frequency of carboxylates (Figure 6, right axis).

The data reported here suggest that in multiply deprotonated gaseous protein ions, [*M*−*n* H]^*n*−^, individual sites are deprotonated according to the gas-phase basicity of the corresponding anions (Table 2). Electron detachment from negatively charged side chains then results in side-chain dissociation, and electron detachment from deprotonated backbone amides results in backbone cleavage into ***a***^•^ and ***x*** fragments. Exceptions from these general observations are radical side chains that are sufficiently long-lived to adopt specific, and in some cases hydrogen-bonded, conformations from which structurally demanding fragmentation into ***c*** and ***z***^•^ fragments can occur. These side chains include those of tryptophan (Scheme 2) and cysteine (Scheme 3). Moreover, the formation of ***c*** ions from cleavage next to Y11, S5, T30, T71, and T137 in EDD of [*M*−23 H]^23−^ ions of BASP1(Δ121–216) (Figure 6) suggests that radical side chains of tyrosine, serine, and threonine may also have adequately long lifetimes to allow for structural rearrangements analogous to those in Schemes 2 and 3 (see Scheme S2 in the Supporting Information). In the absence of acidic side chains (D, E), the probability for backbone amide deprotonation and, therefore, backbone cleavage by EDD increases substantially. Moreover, deprotonation of backbone amides can be facilitated by specific hydrogen bonding to amide oxygen (Scheme 1).

The very same functionalities that make proteins acidic and help bring about high [*M*−*n* H]^*n*−^ ion yields in negative mode ESI, that is, the carboxylates, are largely responsible for reducing the yield of protein backbone fragments in EDD because amide deprotonation is thermochemically less favorable than deprotonation of carboxylic acids. As a result, sequence coverage from ***a***^•^, ***x***, ***c***, and ***z***^•^ backbone fragments was highest for the protein lacking carboxylates, Melittin (72 %), and smallest for the acidic proteins Ferredoxin (12 %) and BASP1(Δ121–216) (19 %); EDD of Ubiquitin gave 63 % sequence coverage. Coincidentally, the relative frequency of carboxylates of the proteins studied here increases (nonlinearly) with their number of amino acid residues. Sequence coverage from ECD of multiply protonated proteins was shown to decrease with increasing number of residues,[2i] and it is reasonable to assume that sequence coverage from EDD of multiply deprotonated proteins is similarly affected by protein size. However, sequence coverage from ECD was 92 % for Melittin,[1b] 97 % for Ubiquitin,[1c] and 65 % for Myoglobin (153 residues, comparable in size to BASP1(Δ121–216) with 147 residues),[1c] which is substantially higher than values from EDD found here, that is, 72 % for Melittin, 63 % for Ubiquitin, and 19 % for BASP1(Δ121–216). Importantly, the decrease in EDD sequence coverage with increasing frequency of carboxylates (from 72 to 19 %) is much steeper than the decrease in ECD sequence coverage with increasing number of residues (from 92 to 65 %). This data corroborates our hypothesis that the major limiting factor for top-down protein sequencing by EDD is side-chain dissociation, in particular, loss of CO_2_ from carboxylates (Figure 6).

The above discussion is limited to top-down sequencing of proteins lacking modified residues, but does our line of reasoning also hold true for EDD of posttranslationally modified proteins? Among the posttranslational modifications that render a protein more acidic are phosphorylation, sulfurylation, and carboxylation. The GB values for phosphate, sulfate and acetate are 1351, 1258, and 1429 kJ mol^−1^, respectively,[23] and thermochemistry predicts that the above modifications carry negative charge in [*M*−*n* H]^*n*−^ protein ions. In analogy to the observed CO_2_ loss from deprotonated glutamic and aspartic side chains ands the C-terminal carboxylate (Figure 1b), electron detachment from phosphate and sulfate modification sites should result in facile loss of these modifications. This supposition is confirmed by EDD spectra of phosphorylated and sulfurylated peptides, which show significant loss of H_3_PO_4_ and SO_3_, respectively.[6a, 7b, 33] However, loss of H_3_PO_4_ in CAD is far more extensive than in EDD,[7b, 33] such that phosphopeptide sequencing is possible by using EDD but not CAD.[33]

## Conclusion

Our study shows that EDD provides sequence information in top-down mass spectrometry of [*M*−*n* H]^*n*−^ ions of acidic proteins consisting of up to 147 amino acid residues. Thermochemical considerations can account for the branching ratio between small-molecule losses and fragments from protein backbone cleavage. Unusual backbone fragmentation into ***c*** and ***z***^•^ fragments can be rationalized on the basis of specific structures, the formation of which requires extended lifetimes of the radical sites involved. Our future studies will focus on strategies to suppress small-molecule losses and increase the yield of fragment ions from backbone cleavage in EDD of acidic proteins.

## Experimental Section

Experiments were performed on a 7 T Fourier transform ion cyclotron resonance (FT-ICR) mass spectrometer (Bruker, Austria) equipped with an ESI source and a hollow dispenser cathode operated at 1.6 A for EDD experiments. ESI flow rate was 1.5 μl min^−1^, and desolvation gas temperature 150 °C. Before ion trapping, precursor ion isolation, and irradiation with 24–28 eV electrons for 100–300 ms in the FT-ICR cell, ions were accumulated in the hexapole ion cells for 0.7–1.5 s. Ion activation prior to EDD and CAD was realized in the second hexapole by energetic collisions with Argon gas. Melittin from bee venom (26 residues, GIGAVLKVLT TGLPALISWI KRKRQQ, with amidated C terminus), mammalian Ubiquitin (76 residues, MQIFVKTLTG KTITLEVEPS DTIENVKAKI QDKEGIPPDQ QRLIFAGKQL EDGRTLSDYN IQKESTLHLV LRLRGG), and Ferredoxin from spinach (97 residues, AAYKVTLVTP TGNVEFQCPD DVYILDAAEE EGIDLPYSCR AGSCSSCAGK LKTGSLNQDD QSFLDDDQID EGWVLTCAAY PVSDVTIETH KEEELTA) were purchased from Sigma (Austria). BASP1(Δ121–216) (147 residues, GGKLSKKKKG YSVNDEKAKD KDKKAEGAAT EEEETPKEAE DAQQTTETTE VKENNKEEKV EKDAQVSANK TEEKEGEKEK TVTQEEAQKA EPEKSEAVVD AKVEPQKNNE QAPKQEEPAS DPSAPPEEAK PSEAPATNSD QTIAVQD) was expressed in *Escherichia coli* cells as outlined in the Supporting Information. Proteins were desalted using centrifugal concentrators as described in reference [2j] and electrosprayed from 1 μm solutions in 1:1 CH_3_OH/H_2_O, with 0.1 or 0.5 % (v/v) 1,8-diazabicyclo[5.4.0]undec-7-en (DBU) as additive. Ferredoxin was denatured during the desalting procedure by using 1:1 CH_3_OH/H_2_O instead of H_2_O, and electrosprayed from solutions that contained ≈50 mm dithiotreitol to prevent disulfide bond formation. For increased statistics, between 250 and 500 scans were added for each spectrum. Ion yields were calculated as percent values relative to all EDD products, considering that backbone dissociation of a parent ion gives a pair of complementary ***a***^•^ and ***x***, or ***c*** and ***z***^•^, ions. Here the even-electron ***x*** and ***c*** ions were used for normalization because their radical ***a***^•^ and ***z***^•^ complements were far less stable (100 %=[***c***]+[***x***]+[other products], in which other products are oxidized molecular ions and losses of small neutral species from oxidized molecular ions).
