# Supplementary material for: Electron Detachment Dissociation for Top-Down Mass Spectrometry of Acidic Proteins
Source: Chemistry. 2011 Mar 23;17(16):4460–9. doi: 10.1002/chem.201003709 (PMC3120980; doi:10.1002/chem.201003709)
Supplement: Supplementary file 1 [file chem0017-4460-SD1.pdf]

CHEMISTRY
A EUROPEAN JOURNAL

Supporting Information

© Copyright Wiley-VCH Verlag GmbH & Co. KGaA, 69451 Weinheim, 2011

Electron Detachment Dissociation for Top-Down Mass Spectrometry of Acidic Proteins

Barbara Ganisl,[a] Taras Valovka,[b] Markus Hartl,[b] Monika Taucher,[a] Klaus Bister,[b] and Kathrin Breuker*[a]

chem_201003709_sm_miscellaneous_information.pdf

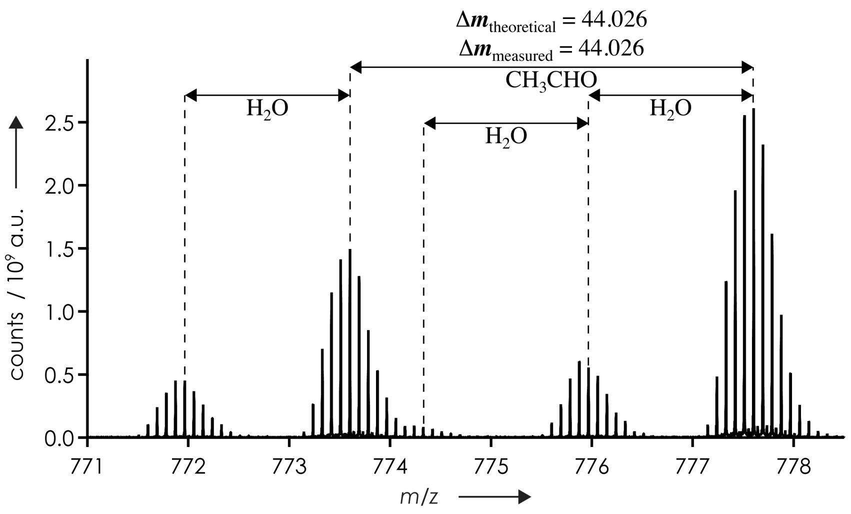

Figure S1. CAD MS spectrum of $(M - 11H)^{11-}$ ions of Ubiquitin from ESI of a 1 μM solution in 1:1 H_2O/CH_3OH, 0.1% Vol. DBU, pH 10.5, illustrating loss of CH_3CHO (44.026) instead of CO_2 (43.990) from $(M - 11H)^{11-}$.

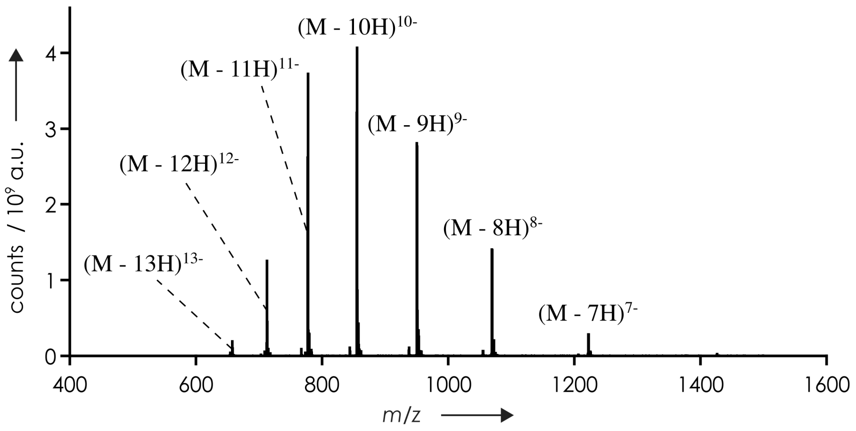

Figure S2. Negative ion mode ESI MS spectrum of Ubiquitin (1 μM in 1:1 H_2O/CH_3OH, 0.1% Vol. DBU, pH 10.5).

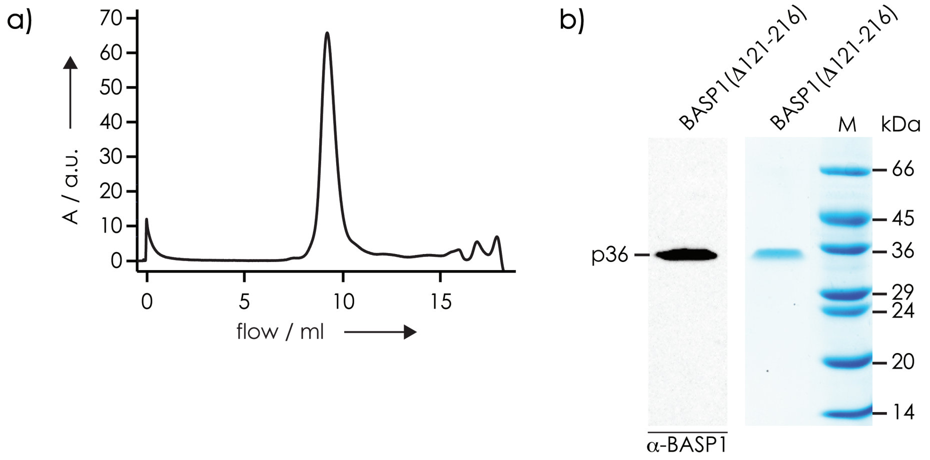

Figure S3. a) Final purification of recombinant BASP1(Δ121-216) protein by size exclusion chromatography on a Superdex 75 10/300 GL column. The protein elutes as a single peak detected by UV light absorbance at 280 nm. b) Analysis of purified BASP1(Δ121-216) by 12.5% (w/v) sodium dodecylsulfate-polyacrylamide gel electrophoresis (SDS-PAGE) (M, marker proteins). Proteins were stained with Coomassie Blue R-250 (right panel), or detected by immunoblotting using polyclonal antibodies directed against a carboxyl-terminal peptide of the chicken BASP1 protein[30] (left panel). The BASP1(Δ121-216) protein displays an apparent M_r of 36,000 (p36). The large deviation from the true M_r is due to the anomalous electrophoretic mobility in SDS-PAGE typically observed for many proteins with low pI.[30,35]

Expression and Purification of Recombinant BASP1(Δ121-216) Protein: The 449-bp *Nco*I/*Bam*HI fragment of pA-BASP1ΔPS[30] containing the coding region for the 148-amino acid BASP1(Δ121-216) protein was inserted into the procaryotic expression vector pET-11d (Novagen), yielding the construct pET11d-BASP1ΔPS. The protein lacks the internal 96 amino acids from position 121 through 216 in the sequence of the full-length 244-amino acid chicken BASP1 protein.[30] BASP1(Δ121-216) has a calculated molecular mass of 16168.867 Da for the most abundant isotope, and an estimated pI of 4.61. DNA of the construct pET11d-BASP1ΔPS was transformed into *Escherichia coli* strain BL21 (DE3) CodonPlus-RIL (Stratagene). To express recombinant BASP1(Δ121-216) protein, bacteria from a single colony were grown overnight at 37°C with shaking at 220 rpm in 10 ml of LB medium containing 50 µg/ml ampicillin and 25 µg/ml chloramphenicol. The bacteria were transferred into 400 ml LB medium containing 50 µg/ml ampicillin, and grown at 37°C with shaking at 220 rpm to an optical density of 0.7 (600 nm). To induce recombinant protein expression, isopropyl-β-D-thiogalactopyranoside (IPTG) was added to a final concentration of 1 mM and bacteria were incubated as above for 4 h. The bacteria were pelleted and resuspended in 25 ml of buffer A (20 mM Tris HCl pH7.5, 80 mM NaCl, 1 mM EDTA, 1 mM DTT, 1 mM PMSF), and then lysed at 1,300 psi using a French Press. *DNAse* I was added to a final concentration of 1.7 µg/ml, and the lysate was incubated at 4°C for 30 min. The sample was centrifuged at 18,000 × g for 20 min at 4°C. Ammonium sulfate was added to the clarified supernatant at 60% (w/v) saturation, and the solution was stirred on ice for 30 min. The precipitated proteins were pelleted by centrifugation at 11,000 × g for 30 min at 4°C. The supernatant was dialyzed for 36 h at 4°C against 2.5 l of buffer A, centrifuged at 18,000 × g for 20 min, and then loaded onto a Mono Q anion exchange column using an automated liquid chromatography system (ÄKTA purifier, GE Healthcare). Chromatography was carried out with a linear gradient from 0 to 0.5 M NaCl in buffer A at a flow rate of 1 ml/min. Fractions containing BASP1(Δ121-216) protein were combined, diluted threefold with dilution buffer (20 mM Tris HCl pH7.5, 1 mM EDTA, 1 mM DTT), and subjected to a second round of anion exchange chromatography. Final elution of the recombinant protein was performed with buffer A containing 280 mM NaCl, followed by ultrafiltration using an Amicon Ultra centrifugal filter device (MWCO 3,000) (Millipore). The concentrated sample was applied onto a Superdex-75 gel filtration column (GE Healthcare) equilibrated with buffer B (50 mM NaH_2PO_4/Na_2HPO_4 pH 7.2, 140 mM NaCl, 1 mM EDTA, 1 mM DTT), and then eluted with the same buffer at a flow rate of 0.5 ml/min. BASP1(Δ121-216) containing fractions were pooled, concentrated and stored at -80°C. The final yield of purified BASP1(Δ121-216) from the 400-ml bacteria culture was approximately 275 µg. ESI MS (with internal calibration, using polyethylene glycol 1000) gave a molecular mass of 16037.814 Da for the most abundant isotope of BASP1(Δ121-216), which agrees with the molecular mass of the predicted sequence exclusive of the N-terminal methionine (16037.827 Da) within experimental error limits (<1 ppm). N-terminal methionine residues are frequently lost by proteolytic processing of proteins expressed in *Escherichia coli*.

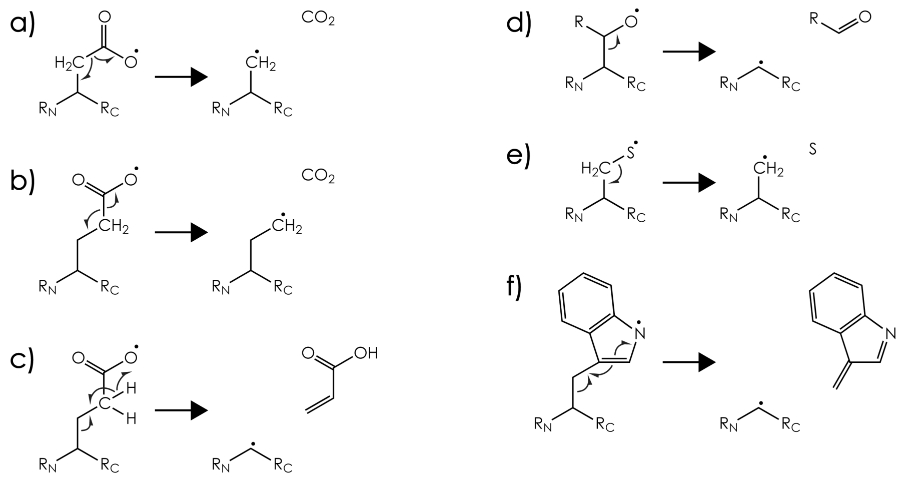

Scheme S1. Proposed mechanisms for small molecule losses from side chains, a) CO_2 from aspartic acid, b) CO_2 from glutamic acid, c) $CH_2CHCOOH$ from glutamic acid, d) RCHO with R = H from serine and R = CH_3 from threonine, e) S from cysteine, f) C_9H_7N from tryptophane; R_N and R_C stand for N-terminal and C-terminal residues, respectively.

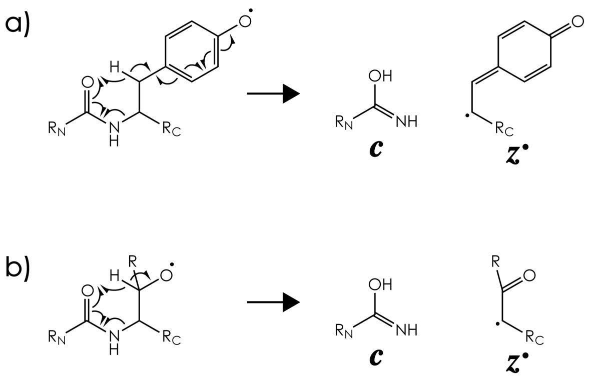

Scheme S2. Proposed mechanisms for ***c*** ion formation by protein backbone cleavage on the N-terminal side of a) tyrosine, and b) serine or threonine (R = H for serine, and R = CH_3 for threonine); R_N and R_C stand for N-terminal and C-terminal residues, respectively.

[35] S. Maekawa, M. Maekawa, S. Hattori and S. Nakamura, *J. Biol. Chem.* **1993**, *268*,13703-13709.
